# Report of the Cholera Conference at Constantinople

**Published:** 1866-08

**Authors:** 


### Miscellaneous.

### Report of the Cholera Conference at Constantinople.

*First Group of Questions—the Origin and Genesis of Cholera; the Endemic and Epidemic prevalence of this Disease in India.*

I—Whence did the cholera, called Asiatic, originally come? And in what countries does it exist in our day in an endemic form?

The Commission with one voice is able to answer without hesitation that the Asiatic cholera, which at different times has run over the whole world, has its origin in India, where it had its birth, and where it exists permanently as an epidemic.

Adopted unanimously.

II—Out of India, does the Asiatic cholera exist in our day in any part of the world in an endemic form?

The Commission considers as demonstrated that the Asiatic cholera, wherever it appears, is never spontaneously developed and has never been observed as an endemic (care must be taken to distinguish secondary foci, more or less tenacious in their character) in any of the countries which have been enumerated, (Europe, etc.,) and that it has always come from abroad. As for the countries in the neighborhood of India, while admitting it as probable that the cholera does not exist there as an endemic, the Commission does not feel itself authorized to come to any formal conclusion on the subject.

Adopted by all the members of the Commission, except MM. Polak, Sawas and Van Geuns.

III—Is there any reason to fear that the cholera may acclimate itself in our countries?

The Commission, without rejecting the possibility of the fact, regards it as problematic.

Adopted unanimously.

IV—Is there in the Hedjaz an original focus of cholera, permanent or periodic?

The Commission is of opinion that Asiatic cholera does not appear to have had in the Hedjaz its original focus, but it appears to have always been introduced there from abroad up to the present time.

Adopted unanimously, except by Mr. Goodeve.

V—Are there in India certain localities which have the exclusive privilege of generating cholera, or which are more particularly favorable to its development? In other words, is cholera endemic in all parts of India, or only in certain regions which it is possible to circumscribe?

At this time the Commission can only answer that there are in India certain localities, comprised principally in the valley of the Ganges, where cholera ls endemic, without being able to point out all of them, or to affirm that they have the exclusive privilege of giving birth to this disease.

Adopted unanimously.

VI—Do we know the causes by the concurrence of which cholera originates spontaneously in India, as well as the circumstances which make it take on an epidemic character?

The Commission feels obliged to limit itself to answering that we know not the special conditions under the influence of which the cholera breaks out in India and reigns there in certain localities as an endemic.

Adopted unanimously.

VII—What are the circumstances which concur in the development and the propagation of epidemics of cholera in India?

The Commission believes itself authorized in answering, that pilgrimages are in India the most powerful of all the causes which tend to develop and propagate cholera epidemics.

Adopted unanimously.

*Second Group of Questions—the Transmissibility and Propagation of Cholera.*

VIII—Is the transmissibility of cholera proved to-day by facts which do not admit of any other interpretation?

Do not all these facts demonstrate conclusively that cholera is propagated by man, and with a rapidity in proportion to the activity and rapidity of his own movements? The Commission does not hesitate to answer in the affirmative.

Adopted unanimously.

The Commission, with unanimity, concludes that the transmissibility of Asiatic cholera is an incontestable verity, proved by facts which do not admit of any other interpretation.

Adopted unanimously.

IX—Are there conclusive facts which force us to admit that cholera can propagate itself at a distance by certain atmospheric conditions, by winds, or by any other change or modification of the surrounding medium?

The Commission answers that no fact has proved, up to the present time, that cholera can propagate itself at a distance by the atmosphere alone, whatever may be its condition; and that besides it is a law, without exception, that never has an epidemic of cholera extended from one point to another in a shorter time than was necessary for man to carry it.

Adopted unanimously.

X—How is the importation of cholera effected, and what are the agents of its transmission?

It may be said, without more specific statement for the moment, that if all modes of conveyance from countries affected with cholera are not likely to propagate the disease, it is none the less prudent, at present, to consider all such means of conveyance as suspected. A more detailed examination will settle the question.

Adopted unanimously.

XI—Under what conditions does man import the cholera?

Man affected with cholera is himself the principal propagating agent of this disease, and a single cholera patient may cause the development of an epidemic.

Adopted unauimously; and—

XII—The Commission has been led to conclude that certain facts tend to prove that a single individual (with much greater reason many individuals) coming from a contaminated place, and suffering from diarrhœa, is able to cause the development of a cholera epidemic; or, in other words, that the diarrhœa called premonitory is able to transmit cholera.

Adopted unanimously.

XIII—What is the period of incubation?

In almost all cases the period of incubation, that is to say, the interval between the moment when the individual may have contracted the cholera poison and the commencement of the premonitory diarrhœa, or of confirmed cholera, does not go beyond a few days; all the facts cited of a longer incubation belong to the class where the contamination may have taken place after departure from the infected place.

Adopted unanimously.

XIV—Can the cholera be imported and transmitted by living animals?

There is no known fact which proves that cholera has been imported by living auimals; but it is reasonable, nevertheless, to consider them, in certain cases, as belonging to the class of objects called susceptible.

A dopted unanimously, except by MM. Bykow and Lenz.

XV—Can cholera be imported and transmitted by linen, clothing, and in geueral by articles in common use!

Cholera can be transmitted by articles in common use coming from an infected place, and especially by those which have been used by cholera patients; and it also results from certain facts that the disease may be transported to a distance by these same articles when closely shut up from the outer air.

Adopted unanimously.

XVI—Can cholera be imported and transmitted by merchandise?

The Commission, while admitting with unanimity the absence of proof of the ageney of merchandise in the transmission of cholera, admits (by a majority of 16 votes to 6) the possibility of the fact under certain conditions.

The negative votes were those of MM. Bykow, Goodeve, Lenz, Pélikan, Polak and Van Geuns.

In consequence, until more fully informed, the Commission believes that it will be wise to consider as suspected, at least under particular and determined conditions, everything coming *(toute provenance)* from a cholera district.

Adopted unanimously, except by MM. Goodeve, Pélikan and Polak, who declined voting.

XVII—Can the bodies of patients who have died of cholera import and transmit the cholera?

Although it is not proved by conclusive facts that the bodies of patients dying with cholera can transmit the disease, it is prudent to consider them as dangerous.

Adopted unanimously, except by M. Sawas, who declined voting.

*On the Influence of Means of Communication.*

XVIII—What influence do the various modes of communication, whether by land or sea, have upon the propagation of cholera?

The Commission answers, that maritime communications are by their nature the most dangerous; that it is they which propagate most surely cholera at a distance, and that next to them comes communication by railroad, which in a very short time may carry the disease to a great distance.

Adopted unanimously.

XIX—What is the influence of deserts upon the propagation of cholera?

The Commission, resting upon facts established by experience, concludes that great deserts are a most effectual barrier to the propagation of cholera, and it believes that it is without example for this disease to be imported into Egypt or Syria, across the desert, by caravans from Mecca.

Adopted by all the members of the Commission, except MM. Monlau, Pélikan, Polak and Van Geuns, who declined voting.

*The Influence of Crowding.*

XX—What is the influence of crowds upon the intensity of epidemics of cholera, as well as upon the propagation of the disease? and under what conditions does it exercise its influence?

All crowding together of human beings, among whom cholera has been introduced, is a favorable condition for fhe rapid spread of the disease—and, if this crowding exists under bad hygienic conditions, for the violence of the epidemic among them.

That in this case the rapidity of the extension of the disease is in proportion to the degree of crowding, while the violence of the epidemic is, other things being equal, so much the greater according as individuals have been little exposed to the choleraic influence or not at all; that is to say, in other words, individuals who have already been exposed to the influence of a cholera atmosphere enjoy a sort of relative and temporary immunity which counterbalances the bad effects of crowding.

Finally, in the case of a dense crowd, the more rapid its separation, so much the more rapid is the cessation of the epidemic, at least if new arrivals of unaffected persons do not furnish new aliment for the disease.

Adopted unanimously.

XXI—What is the intensity and what the tenacity of cholera epidemics on shipboard?

The Commission replies that the intensity of cholera on board ships crowded with men is, in general, proportionate to the crowding, and is so much the more violent, other things being equal, if the passengers have not resided in the focus of cholera which they started; that on crowded ships the spread of cholera epidemic is ordinarily rapid; finally, the Commission adds that the danger of importation by ships, and that of giving rise to a grave epidemic, are not entirely subordinate to the intensity, nor even to the existence of choleraic symptoms appearing during the voyage.

Adopted unanimously, except by M. Monlau, who declined voting.

XXII—What influence does the accumulation in lazarettos of individuals coming from a cholera district exercise upon the development of cholera among the people at quarantine and in the neighborhood?

The Commission concludes that the crowding together of people coming from a place where cholera reigns in a lazaretto, has not the effect of producing, among the people at quarantine, a great extension of the disease; but that such a gathering is nevertheless very dangerous for the neighborhood, as it is calculated to favor the propagation of cholera.

Adopted unanimously, except by M. Monlau.

XXIII—What influence do great collections of men, in armies, fairs, pilgrimages, exercise upon the development and propagation of epidemics of cholera?

The Commission concludes that great gatherings of men (armies, fairs, pilgrimages) are one of the most certain means for the propagation of cholera; that they constitute the great epidemic foci which, whether they march after the manner of an army, or whether they are scattered, as at fairs and in pilgrimages, import the disease into the country which they traverse; that these gatherings, after having been exposed, usually in a rapid manner, to the influence of cholera, become much less susceptible to its power, and that it disappears very speedily, unless newly arrived persons take the disease.

Adopted unanimously.

XXIV—What is the influence of dissemination upon the intensity and development of cholera epidemics?

The Commission concludes that the breaking up of a collection of people, at an opportune time, may render less violent an epidemic of cholera and even arrest its extension; but that this scattering, on the other hand, gives rise to great danger of propagating it, if it take place in the midst of a region as yet unaffected.

Adopted unanimously.

XXV—What part belongs to the pilgrimage to Mecca in the cholera epidemics of our day.

The part of the pilgramage to Mecca, as an agent in propagating cholera as regards the neighboring countries of Europe (the only one with regard to which we have positive information) has been the introduction of this disease into Egypt twice, with an interval of thirty-four years, during the hot season.

Adopted unanimously, except by M. Polak, who declined voting.

*The Influence of Hygienic Conditions.*

XXVI—What is the influence upon the violence of cholera epidemics exerted by hygienic and other conditions of locality; in other words, what are the assisting causes of cholera?

The Commission recognizes that the hygienic and other conditions which in general predispose a population to contract cholera, and consequently favor the intensity of epidemics, are: misery, with all its consequences; overcrowding, particularly of persons in feeble health; the hot season; want of fresh air; the exhalations from a porous soil impregnated with organic matters, above all with the dejections from cholera patients.

In addition, the Commission think that, as it appears demonstrated by experience that the discharges of cholera patients contain the generative principle of cholera, it is right to admit that drains, privies, and the contaminated waters of towns may become the agents for the propagation of this disease.

The Commission adds, that it seems to result from certain facts that the soil of a locality, once impregnated with cholera detritus, is able to retain for a considerable length of time the property of disengaging the principle of the disease and of thus keeping up an epidemic, or even of regenerating it after it has become extinct.

Adopted unanimously, except by M. Pélikan.

*Immunity from Cholera.*

XXVII—How is immunity from cholera to be interpreted?

The immunity which certain localities enjoy, that is to say, the resistance, permanent or temporary, general or partial, opposed by these localities to the development of cholera within their limits, is a fact which does not exelude transmissibility, but which indicates that certain local conditicns, not yet entirely determined, are an obstacle to the development of the disease.

The same immunity, more or less complete and more or less durable, which the majority of persons in the midst of an infected district enjoy, an immunity which attests the individual resistance to the toxic principle, is a circumstance to which we should attach the highest importance.

In point of view of epidemic development, it is the corrective of transmissibility, and viewed with regard to prophylaxia, it sets in operation proper means to arrest the ravages of the disease.

Adopted unanimously, except by MM. Monlau and Pélikan, who declined voting.

*Deductions relative to the Generative Principle of Cholera.*

XXVIII—From the facts above established, and which relate to the genesis, the propagation and the transmissibility of cholera, can we draw any precise conclusion with regard to the generative principle of the disease, or at least wiih regard to the media which serve as its vehicles, or receptacles; with regard to the conditions of its penetration into the organism, the ways by which it passes out, the duration of its morbific activity, in a word, with regard to all its attributes, a knowledge of which is important to guard against it?

In the actual state of science, we can only frame hypotheses as to the generative principle of cholera; we know only that it originates in certain countries of India, and that it dwells there permanently; that this principle is reproduced in man and accompanies him in his journeyings; that it may also be propagated at a distance, from place to place, by successive regenerations, without ever being reproduced spontancously outside of man.

Adopted unanimously, except by M. Goodeve, who declined voting.

XXIX—What are the vehicles of the generative principle of cholera?

Under the name of vehicles, the Commission intends to speak merely of the agents by means of which the morbific principle penetrates the organism. To this question the facts reply that the air is the principal vehicle of the cholera principle. * * * The action of the cholera miasm is so much the more sure as it operates in a confined atmosphere and near the focus of emission. * * * * It seems that it is with cholera miasm as it is with the miasm of typhus, which rapidly loses its power in the open air at a short distance from its starting point.

XXX—To what distance from a focus of disease can the principle of cholera be transported by the atmosphere?

The surrounding atmosphere is the principal vehicle of the generative agent of cholera; but the transmission of the disease by the atmosphere, in an immense majority of cases, is limited to a space very near to the focus of emission. As for the facts cited of transportation by the atmosphere to the distance of one or more miles, they are not sufficiently conclusive.

Adopted unanimously, except by M. Goodeve, who declined voting.

XXXI—Independent of the air, what other vehicles are there of the cholera principle?

Water and certain ingesta may also serve as vehicles for the introduction into the organism of the generative principle of cholera.

This granted, it follows, so to speak, necessarily, that the passages by which the toxic agent penetrates into the economy are principally the respiratory passages and very probably also the digestive canals. As for its penetration by the skin, nothing tends to prove it.

Adopted unanimously.

XXXII—What are the principal receptacles of the cholera principle?

The matter of the cholera dejections being incontestably the principal receptacle of the morbific agent, it follows that everything which is contaminated by the discharges become also a receptacle from which the generative principle of cholera may be disengaged, under the influence of favorable conditions; it follows, also, that the origin of the cholera germ takes place very probably in the digestive canal, to the exclusion, perhaps, of all other parts of the system.

Adopted unanimously.

XXXIII—What is the duration of the morbific activity of the generative principle of cholera?

It results from the study of facts, that in the open air the generative principle of cholera loses rapidly its morbific activity, and that this is the rule; but that under certain particular conditions of confinement, this activity may be preserved for an unlimited period.

Adopted unanimously.

Finally, the Commission adopts the following formula:

Observation shows that the duration of the choleraic diarrhœa, called premonitory—which must not be confounded with all the diarrhœas which exist during the time of cholera—does not extend beyond a few days.

Facts cited as exceptional do not prove that the cases of diar rhœa prolonged beyond that period belong to cholera, and are susceptible of transmitting the disease, when the individual affected has been withdrawn from all cause of contamination.

Adopted by fourteen votes against four, viz: MM. Gomez, Millingen, Mühlig and Salvatori; M. Monlau declined voting.

Here end the labors of the commission, with regard to the origin, the endemic condition, the transmissibility and the propagation of cholera, and the historic sketch of the march of the epidemic of 1865, made by a sub-committee, of which Dr. Bartoletti was the Secretary, before being presented separately to the conference.

With regard to the different questions placed upon the programme, it is to be said, that by limiting themselves to drawing from facts the consequences which reasonably flow from them, the Commission thinks it has established sure foundations which will enable the conference to pronounce understandingly upon all questions relating to prophylaxia.

Signed by A. FAUVEL, Sec'y.

The present report, having been discussed and adopted, chapter by chapter, was approved as a whole by all the members of the Commission.

*Constantinople, May* 21, 1866.

Signed by all the members of the Commission.

The above abstract gives, in a condensed form, the substance of a report which confirms in the strongest manner all that this and other journals in this country and abroad have maintained with regard to the communicability of cholera. It is not strange, therefore, that, as is stated by the French press, the Conference adopted the following propositions, presented by the French delegates, as we learn from the *Medico-Chirurgical Review:*

"To break off all communication—the moment cholera appeared among the pilgrims—between the Arab ports and Egyptian coast, leaving the land route followed by the caravan open for the hadjis for their return to Egypt. In other words, the pilgrims would be obliged to perform quarantine, either in the Hedjaz till the epidemic ceased, or in the desert in the caravan route."—*Boston Medical and Surgical Journal.*

